# Exploring the Relationship between Housing and Health for Refugees and Asylum Seekers in South Australia: A Qualitative Study

**DOI:** 10.3390/ijerph14091036

**Published:** 2017-09-08

**Authors:** Anna Ziersch, Moira Walsh, Clemence Due, Emily Duivesteyn

**Affiliations:** Southgate Institute for Health, Society and Equity, Flinders University, Adelaide 5042, Australia; moira.walsh@flinders.edu.au (M.W.); clemence.due@flinders.edu.au (C.D.); eduivesteyn@hotmail.com (E.D.)

**Keywords:** health, wellbeing, housing, asylum seeker, refugee, humanitarian migrant

## Abstract

Housing is an important social determinant of health; however, little is known about the impact of housing experiences on health and wellbeing for people from refugee and asylum-seeking backgrounds. In this paper, we outline a qualitative component of a study in South Australia examining these links. Specifically, interviews were conducted with 50 refugees and asylum seekers who were purposively sampled according to gender, continent and visa status, from a broader survey. Interviews were analysed thematically. The results indicated that housing was of central importance to health and wellbeing and impacted on health through a range of pathways including affordability, the suitability of housing in relation to physical aspects such as condition and layout, and social aspects such as safety and belonging and issues around security of tenure. Asylum seekers in particular reported that living in housing in poor condition negatively affected their health. Our research reinforces the importance of housing for both the physical and mental health for asylum seekers and refugees living in resettlement countries. Improving housing quality, affordability and tenure security all have the potential to lead to more positive health outcomes.

## 1. Introduction

People from refugee and asylum-seeking backgrounds represent some of the most marginalised and vulnerable groups in the world [1,2,3,4] facing a range of risk factors for poor health and wellbeing, including experiences of trauma, dislocation and violence, with associated loss of family and community support and ontological insecurity (e.g., see [1,2,5,6,7,8,9,10,11]). When resettling in new countries, there are a range of factors that are important for successful health outcomes, such as finding employment, engaging in education, building social connections and accessing services. Amongst these factors, housing is of key importance, and secure housing is a human right and an important social determinant of health [12,13,14,15,16,17,18,19,20,21,22,23,24]. In relation to refugees and asylum seekers, access to appropriate housing is a marker of successful integration, as well as a means through which those people who are newly arrived can build a new life in host countries [25,26]. Indeed, given the psycho-social factors associated with conditions of displacement, finding and holding on to appropriate housing can be the most critical indicator of successful integration for refugees and asylum seekers [25,26,27].

However, there is currently little research that clearly outlines the links between housing and health outcomes for asylum seekers and refugees in countries of resettlement such as Australia. Correspondingly, in this paper, we explore the links between housing and health for people from refugee and asylum-seeking backgrounds drawing on in-depth interviews with 50 people resettled in South Australia, Australia. We examine the extent to which people felt that housing had an impact on their health and wellbeing, which aspects of housing were implicated in relation to health and wellbeing, and whether there were any differences amongst the group on the basis of demographic criteria such as visa status and family size.

### 1.1. A Note on Terminology

It is important to acknowledge the heterogeneous experiences and identities encompassed in the terms “asylum seeker” and “refugee”. Asylum seekers are defined as people who are outside their country of origin and seeking formal protection, but have not had their claims to refugee status assessed [28,29,30]. In contrast, refugees are defined as people who meet the criteria for refugee status, typically as defined by the United Nations High Commissioner for Refugees (UNHCR), but sometimes as defined by particular criteria outlined by specific countries [31]. Importantly, after resettlement, people may also no longer feel these terms apply to them. Thus, it is acknowledged that these terms are problematic in subsuming complex and diverse identities and lived experiences into single categories.

We draw on the World Health Organization (WHO) definition of health as “a state of complete physical, mental and social wellbeing and not merely the absence of disease or infirmity” [32] (p. 100).

### 1.2. Approaches to Considering Housing as a Social Determinant of Health

The World Health Organization (WHO) defines social determinants of health (SDOH) as the “conditions in which people are born, grow, work, live, and age, and the wider set of forces and systems shaping the conditions of daily life” [12]. Housing is one of these determinants, and a growing body of research has indicated that housing has a significant effect on overall levels of wellbeing and specific mental and physical health outcomes such as depression and respiratory illnesses [12,13,14,15,16,17,18,19,20,21,22,23,24]. In addition, research indicates that housing and health share a complex relationship both in terms of health outcomes at an individual level and in relation to health inequalities at a population level [16,33,34] and that effects are likely to be both direct and indirect [21]. Similarly, these relationships are likely to be bi-directional as poor mental and/or physical health, together with other social factors such as education, race and ethnicity and wealth, are known to significantly impact one’s ability to secure appropriate housing [15,35,36,37,38].

Adequate housing is enshrined as a human right [39], and the WHO argues that “adequate” means more than just a physical shelter, but “to have a home, a place which protects privacy, contributes to physical and psychological wellbeing and supports the development and social integration of its inhabitants” [40] (p. 413). The WHO urges a four-layer model of housing taking into consideration not just the physical structure of the house, but also the meaning of home and the social and physical features of the neighbourhood. Each of these features is seen as interlinked, and all have the potential to affect health and wellbeing both individually and in combination. The WHO suggests eight key goals in addressing housing as an SDOH where housing should have sound construction, provide safety and security, be of adequate size, offer basic services such as clean water and sanitation, be reasonable and affordable, accessible to services and amenities, offer reasonable security of tenure and provide protection from climate changes [12].

Baker and colleagues have considered the relationship between housing and health in Australia from a social determinants of health perspective (e.g., see [15,34,41]). Their research takes the view that in order to accurately depict the health impacts of housing and enable more directed policy recommendations, housing issues or problems (particularly affordability, condition, tenure, location) should be viewed as a collection of components, rather than as separate problems. In relation to the Australian context, they note the high standard of housing relative to other parts of the world, but argue that there is a “hidden fraction” of Australians who are living in housing that is in poor condition and that negatively impacts their health and wellbeing (e.g., [36,42]).

Following this cumulative approach to housing problems and the impacts on health is Mallett and colleagues’ [36] conceptualisation of precarious housing. Specifically, they suggest that precarious housing comprises at least two of the three following elements. They argue that housing can influence health to the extent that it is suitable (including the hardware or physical features of the house, the space in terms of crowding and privacy and the location in terms of the neighbourhood physical features such as green spaces and street lighting and social environment), affordable and offers secure tenure. They propose a number of intermediary factors between housing and health—identity, stability, safety, social support, physical environments, living practices and sense of control, and argue that these relationships between housing and health are also mediated by age, gender, culture and personal circumstances. Evidence for this model and the components of housing suitability, affordability and security has been found in a recent synthesis of research conducted with the general Australian population [43].

Importantly, neither Baker and colleagues’ discussion of housing in Australia, nor Mallett and colleagues’ conceptualisation of precarious housing and health were developed specifically for the refugee and asylum seeker context. Ager and Strang’s model of integration [25,26], which considers the broad range of factors affecting the integration of refugees and asylum seekers, offers further insight into the potential links between housing and health for these groups. This model includes ten policy indicators of integration that are organized into four separate though interconnected domains. Housing belongs to the first domain—“Means and Markers”—along with employment, education and health. Each of these elements are seen as markers of successful integration, as well as a means to achieving integration given that they can support the advancement to other facets. The domain “Social Connections” takes account of the concept of social capital and Putnam’s [44] three dimensions of social bonds (between members of own community), bridges (ties to other communities) and linkages (links to wider social institutions). The third domain, “Facilitators” includes language and cultural knowledge and safety and stability, which support refugees to inhabit their neighbourhood and connect to their community with a sense of confidence. The last domain, “Facilitators” deals with the rights of refugees, others and the state with a particular emphasis on the obligations and expectations associated with citizenship.

Within this model, secure housing can be seen as a key marker of integration that impacts on health for refugees and asylum seekers [27]. In tandem with a cumulative approach to housing problems and consideration of the precarity of housing, aspects of integration add another important component to the relationship between housing and health and the interlinkages with other aspects of resettlement. 

In this paper, we use these models together with a social determinants of health approach in order to consider the link between housing and health for refugees and asylum seekers resettling in South Australia.

### 1.3. Housing and Health for Refugees and Asylum Seekers

Despite the provision of some housing in initial stages of resettlement in many countries (including Australia), previous research indicates a range of housing issues for refugees and asylum seekers, including the cost of housing, experiences of discrimination, lack of available or appropriate housing stock, language barriers and a lack of familiarity with housing and sociocultural elements of resettlement countries [5,35,45,46,47,48,49,50,51,52,53]. In Australia specifically, research has indicated that over 40% of refugees may have difficulty finding somewhere to live [54], while a separate study found that 75% of respondents indicated that the cost of rental accommodation (in which the majority of refugees are housed) was a challenge [35,48]. Other challenges have included physical elements such as poor condition of housing or overcrowding [55], as well as issues in social aspects of housing such as safety and cultural appropriateness [56] and discrimination [45,57].

There are relatively few studies concerning housing and health for asylum seekers and refugees. What is available suggests that this cohort may be at particular risk of health-related outcomes as a result of housing experiences. For example, a study of refugees living in Switzerland [58] found that rates of post-traumatic stress disorder (PTSD) were higher amongst people who did not have family members or friends to live with, while research in the UK [59] has found that poor housing conditions (such as dampness and mould) lead to high rates of mobility, which in turn increased negative mental health outcomes. In the U.S., unstable housing has been linked to severely impaired global functioning [11], while overcrowding has also been associated with negative health outcomes [60]. Insecure housing has also been linked to negative mental health outcomes particularly for women, as a result of safety concerns [61]. In Australia, research by Fozdar [57] found that mental health outcomes amongst people with refugee backgrounds was primarily connected to material and social factors, which included housing. A qualitative study of 12 refugee women in Sydney found links between difficulties securing housing (particularly for large families) and mental health [50]. Finally, in a paper exploring Ager and Strang’s model of integration, Phillimore and Goodson [27] note that several of their studies point to the potential bi-directional relationship between housing and health, specifically noting that refugees experiencing health problems were negatively impacted in their ability to find appropriate housing.

Several studies have indicated that refugees themselves identify housing as a social determinant of health. For example Carter, Polevychok and Osborne [47] found that 30% of their sample of refugees felt that housing contributed to health problems in their first year of resettlement in Canada, while Papadopoulos and colleagues [61] found that 37% of their sample attributed ill-health in part to their housing circumstances in the UK Fozdar and Hartley [2] found that participants perceived a negative relationship between the quality of their housing upon arrival in Australia and their health outcomes. Moreover, research by Fennelly [62] with service-providers in the U.S. indicated a belief that instability in housing and overcrowding led to difficulties managing physical health needs such as tuberculosis. Palmer and Ward [63] and Palmer [64] both found that inappropriate housing or homelessness was cited as one of the main reasons for poor mental health outcomes for refugees in the UK.

As highlighted above, there is limited research concerning the link between housing and health for refugees in Australia with its particular housing environment [2,50,57] and none that has specifically considered the experiences of asylum seekers. As such, this paper seeks to draw upon Mallett and colleagues’ [36] cumulative approach to examining housing problems and their impacts on health, in concert with Ager and Strang’s framework of integration [25,26] through a focus on housing as a key social determinant of health and as a means to successful integration for people from refugee backgrounds. Specifically, it will explore the relationship between housing (as a means and marker of integration) and the self-reported health and wellbeing of asylum seekers and refugees.

## 2. Materials and Methods

The results outlined in this paper form part of a larger study of the relationship between housing, social inclusion and health for asylum seekers and refugees who had been in Australia for seven years or less. Specifically, this study involved a survey of over 400 people who arrived in Australia as asylum seekers or refugees. People who participated in this survey could then elect to participate in an interview. These interviews form the data for the current paper.

Participants in the study who were refugees would have been eligible for general settlement support provided for their first six months (sometimes 12 months) in Australia, including: help with transport to initial accommodation upon arrival, provision of initial accommodation (typically for a period of 6 months, although this is variable), assistance with finding longer term accommodation, property inductions, orientation to life in Australia and other case support. People from asylum seeker backgrounds would have received assistance with accommodation for approximately six weeks.

### 2.1. Participants

Semi-structured interviews were conducted with 50 participants, including 28 people from refugee backgrounds (i.e., had permanent protection visas) and 22 asylum seekers (who were on temporary visas awaiting the determinant of their claim for refugee status), who were purposively sampled from participants in the broader survey to include people from a range of cultural backgrounds, visa status and also gender. Of the refugees, 12 were women and 16 men, while 10 came from Africa, nine from the Middle East and nine from South East Asia. Of the asylum seekers, 10 were women and 12 men, with all coming from the Middle East. In this paper, we use pseudonyms to describe participants and include their gender, continent and visa status for any direct quotes we use.

### 2.2. Procedure and Data Analysis

Ethics approval for the study was obtained from the Flinders University Social and Behavioural Ethics Committee (project 6273), and the researchers also complied with a range of other ethical considerations imperative to working with refugees and asylum seekers who can broadly be considered a “vulnerable” group for the purposes of research [65]. This included particular attention to issues of coercion and informed consent given potential cultural norms of agreeing to participate in research, potential power imbalances between researchers and participants, as well as assurances of confidentiality and anonymity and that service access was not related to participation. Project documentation was translated into key language groups for participants, and interpreters were available. Furthermore, the project was conducted with the assistance of a refugee and asylum seeker working group to ensure that the project was conducted in partnership with communities. Informed consent was gained from all participants.

Interview participants were recruited through the original survey, which included a question asking people if they were interested in participating in a follow-up interview. The researchers contacted participants by telephone. Where participants had indicated in the survey that their level of English was not strong, they were contacted with the assistance of an interpreter in the language in which they completed the survey (Dari, Farsi, Swahili, Nepali or Arabic). Participants were told they did not have to participate in an interview if they had changed their minds. Where they still wished to participate, a time and place was organised to meet face to face at their preferred interview location, with an interpreter if the participant chose. The interviews lasted up to 70 minutes, with an average of 32 min.

Interview questions were developed in response to the literature outlined in the previous section concerning housing and health for refugees and asylum seekers. Specifically, interviews included questions about participants’ housing journey in Australia, what they looked for in housing, how they had been able to find housing in the past, whether they were satisfied with their housing in Australia and whether they felt their housing impacted upon their physical and/or mental health.

The data were thematically analysed using the framework approach [66], which involves five key stages. Familiarisation involved being immersed in the data, by reading and re-reading interview transcripts and listing key ideas and recurrent themes. The thematic framework was devised by considering the original research aims, the interview schedule, the issues identified by the interview respondents, as well as the recurring views and experiences noted during familiarisation. Indexing was achieved by coding all of the interview transcripts using the NVivo Version 10 qualitative software database (QSR International, Melbourne, Australia), according to the thematic framework. In the charting phase, a series of thematic matrices was developed charting each participant against the emergent themes. In the final mapping and interpretation phase, people’s housing experiences were outlined, variations between different contexts and groups identified, and explanations for these developed.

## 3. Results

In this section, we outline the general links that participants made between housing and health and wellbeing, as well as discuss the impact of specific elements of housing that emerged from the analysis: housing affordability, the physical elements of housing such as cold and damp and space and layout, social elements of housing such as safety and disorder and social relations and insecurity of housing tenure.

### 3.1. Overall Health and Wellbeing

Many asylum seekers and refugees have suffered a range of human rights violations, prolonged exposure to trauma and torture so that when they arrive in Australia, they often present with complex health issues [10,67,68,69,70]. Amongst the participants of this study, a significant number indicated health issues particularly in relation to mental health. Respondents described feelings of loneliness, worry about family who were not in Australia, visa status, housing circumstances and financial situation. These feelings of worry variously manifested as self-described symptoms of anxiety, depression and stress, and for a small number, suicidal ideation. In relation to physical health, respondents most commonly reported chronic pain and sleep disturbances.

While participants noted that there were other significant factors at play (e.g., visa status and income levels), the accounts of both refugees and asylum seekers revealed that housing was a central aspect of their health. Several respondents suggested the simple fact of having a house was important for mental health, as seen in the following quotes:

When we have a home we live in peace of mind and relax without problems.

—Bijan (male, Middle East, asylum seeker)

Everybody needs accommodation to feel safe in it, which is very important. If you had a place then you’ve got peace of mind and that helps to reduce your anxiety.

—Mina (female, Middle East, refugee)

For those who felt housing was not so significant for their health, this related to the relative impact of other factors (visa status, securing employment and family separation) that were seen as greater. For others, problems with housing were seen as having a compounding impact on health and wellbeing.

While simply having a home could inspire a sense of wellbeing for this cohort, a range of additional housing-related factors was seen by participants as important to health. Specifically, for the participants of this study, housing impacted on health through a range of pathways. This included affordability, the suitability of housing in relation to physical aspects such as poor condition and problems with layout, issues around security of tenure and social aspects of housing linked to feelings of safety, belonging and privacy. Importantly, these factors did not exist in isolation, and cumulative health and wellbeing impacts were noted where people were experiencing issues in more than one element of their housing.

### 3.2. Affordability

The cost of living (e.g., accommodation and bills) was considered a fundamental issue for almost all of the participants of this study. In particular, the cost of rent was identified as a key measure of housing satisfaction with the vast majority finding that rent was too expensive. This was particularly the case for asylum seekers. In Australia, asylum seekers are typically on bridging visas, which allow them to remain in the country in the short term while their claims are assessed. Those on bridging visas often find it hard to find employment due to the short-term nature of their visas and are only eligible for 89% of the standard social security payments. Where an asylum seeker has their claim denied twice, they are often cut off from all financial assistance.

Concerns around the affordability of suitable housing is seen in the following quote from Aaron, a single male asylum seeker who has been sharing with several other new arrivals. Rather than the overcrowded conditions in which he is living, his preference would be for a place of his own:

I like to have that kind of place (own room) but I cannot afford it. The problem is financial problem... Because I have a language barrier and I don’t have a job, when I go and apply for a job usually they ask me my language and when I said I don’t speak English, then they won’t give me job; as a result at this age I cannot get the job. If I can find a job and make money then I can afford to get a better place, otherwise with the government support I cannot afford to get a better place. Because of the financial difficulties, that put me under pressure that live with sharing the house and room with others.

—Aaron (male, Middle East, asylum seeker)

Aaron makes explicit the links between poor English language skills, the ability to find work and the ability to afford suitable housing, which would have a more positive impact on his overall wellbeing. The absence of key facilitators of integration (language and stability in relation to his migration status) acts as a barrier to Aaron’s ability to access the key means of integration (education and housing). Moreover, Aaron’s housing situation clearly points to issues related to insecure tenure, further exacerbating his precarious housing situation.

The cost of living for some of the larger families in the study (who were frequently from an African country) also had some negative impacts on mental health in relation to stress and worry. For example, Naeva and Daina, both African, from refugee backgrounds and with six and seven children, respectively, say:

The house is very expensive; this disturbs me a lot.

—Naeva (female, Africa, refugee)

The house is too old and expensive, sometime my children ask me mum why are we living in this house? It is difficult in Australia who have big family to have good house.

—Daina (female, Africa, refugee)

In addition to cost, Daina (above) describes her house as “too old,” further suggesting the difficulties large families face in finding suitable housing that is in good condition.

### 3.3. Physical Elements

Mallett and colleagues refer to the hardware or physical elements of a dwelling and the space in terms of crowding and privacy, which all contribute to the suitability of a particular house. Reflecting on these factors, all participants described physical elements as important, although different participants discussed a variety of aspects as unsatisfactory or undesirable. Of note across the interviews was the overall poor condition (e.g., lack of heating and or cooling, mould, dampness, peeling paint, leaking pipes and ceilings, broken amenities, damaged blinds, overgrown gardens) and a lack of space or unsuitable layout (e.g., small rooms, not enough bedrooms and bathrooms, no yard). Some participants noted that there was an association between these various physical factors and affordability. For example, Samuel, a refugee from Africa, stated:

Housing affordability is an issue that affect diverse and linguistic people as we do not have referees and this cause most refugees and Asylum seekers to sign contracts for houses that are in bad condition.

—Samuel (male, Africa, refugee)

As Samuel notes, housing that is in poor condition is often the only affordable choice available to asylum seekers and refugees. Unsuitability in terms of poor condition and unaffordability reflect the precarious nature of housing for people from refugee and asylum seeker backgrounds, which result in a range of impacts on health and wellbeing. According to the participants of this study, cold and damp housing, overcrowding and lack of space had the greatest impact on health and wellbeing, and this was particularly significant for mental health. These are discussed in turn below.

#### 3.3.1. Cold and Damp

Cold and damp housing was found to be one of the most significant factors highlighted across interviews as causing a range of physical and mental health-related issues such as respiratory problems, sadness/depression and joint pain.

*(The house) It’s kind of wet. It’s kind of moisture, yeah, and as a result of that most of the time we feel the chronic pain, like a lower back on the foot and those kinds of things.*

—Aaron (male, Middle East, asylum seeker)

Notwithstanding the physical and mental impacts on health associated with cold, damp and mouldy living conditions, having a sense of control over issues—such as those noted in the quotes above—is a key intermediary between housing and health [36]. For those experiencing these problems, there was a perceived difficulty in addressing housing concerns. This was particularly the case for those from asylum seeking backgrounds, where the sense of a lack of control appeared amplified by their precarious visa status and concern for breaching any conditions associated with this. For example, Sajad, an Iranian asylum seeker living in a private rental with his sister and her husband, noted that he felt frustrated that their landlord refused to do repairs. Sajad indicated that since he and his family were refugees (in his case, still awaiting determination of their claim), they did not feel that they could address the issues:

Some house very (mouldy), make bad for breathe or something, chest (…) now, inside my house very cold than outside (…) but I cannot say very bad because we are refugee here and we cannot explain.

—Sajad (male, Middle East, asylum seeker)

Those participants with permanent visas similarly also noted that they felt that cold, damp and mould impacted their physical health and that they were living in poor housing because of their refugee status. However, at least for some with permanent protection, there appeared a greater sense of agency compared to asylum seekers to raise the issues with landlords. For example, in contrast to asylum seeker Sajad (above), Edris, who had newly arrived from the Middle East with a permanent protection visa, felt able to express his concerns:

It was winter and the house became cold and green—with the green stuff—mould and we know the mould can cause some breathing—breathing—asthma (…) I was a little bit sad, and a little bit angry too (about the poor condition of the house) I said to (the caseworker)—yes It’s true—I come from the Middle East–but still I know my rights

—Edris (male, Middle East, refugee)

Despite this, Edris and his family were advised that all of the housing that was affordable to them would have similar issues with condition.

Problems with housing condition relating to cold, dampness and mould in the house reflect two aspects of Mallett and colleagues’ elements of precarious housing, namely unsuitable housing and insecurity of tenure. Importantly, insecurity of tenure was associated with the participants’ rental status and visa status, which both contribute to the absence of control over housing issues, and the adverse effect these issues have on physical and mental health and wellbeing.

#### 3.3.2. Space and Layout

An unsuitable layout (e.g., size, too few rooms, having stairs) was another aspect of the physical features of housing identified by some participants as having an impact on health and wellbeing. Space and layout were generally related to specific housing situations faced by some of the people in interviews. Overcrowded share-houses, for example, were seen as negatively related to mental health, particularly for single male asylum seekers due to lack of space, privacy and incompatibility with housemates. For example, Adeeb, an asylum seeker from Afghanistan on a bridging visa, had shifted several times in the four years he has been in Australia, living in share houses and with family friends. In his interview, he noted the negative impact on mental health of these forms of housing:

So last year or before last year, I think in 2014, I got a mental problem as well because we knew that the house is very noisy but (…) they don’t care about people like that. If you’re making the food, you cook the food anyone can eat the—(they never let) no other people to come to dinner with them. Yeah it’s a little bit (difficult).

—Adeeb (male, Middle East, asylum seeker)

In addition, participants with large families also cited space as an issue, with their housing providing inadequate room for their family members. These problems are evidenced in the account of Nafisa, an asylum seeker from Afghanistan who found her overcrowded housing negatively affecting her health, compounded by a range of other issues (e.g., cost of housing, lack of references, changing children’s school) that make it difficult for her to improve her living circumstances. Exacerbating these problems is the absence of social “bridges” with her neighbours who she describes as unhappy about her children playing in the units’ shared carpark. She says:

Yes, actually it does affect me a lot. You know, the problem is I can’t sleep at night time, I can’t sleep well, it’s because of having not good house (…) the house is very small. It’s not good enough for four people (…) my kids, they can’t go outside. We don’t have back yard, they can’t play outside. The house is very small for them to play.

—Nafisa (female, Middle East, asylum seeker)

Nafisa has been unable to find alternative housing, and she is resigned to staying where she is, which to some extent is mediated by her landlord, about whom she says: “the only reason we live in this house is our owner; the owner of the house is a very good person. He’s a very, very nice person.”

The circumstances of Asmita, a refugee from Southeast Asia, reflect the impact of overcrowding for large families on mental health from a young person’s perspective. Asmita lives with her parents and siblings and struggles to gain the privacy and space that she requires for her wellbeing:

When your house is small and you are sharing your bedroom and there is no place for you to get out and be alone and give time for yourself, at that time when you want to come out of that down situation and you don’t have that place to be, or place to go, and yeah it has really affected me.

—Asmita (female, Southeast Asia, refugee)

Elsewhere in the interviews, housing that was considered clean and in good condition was described as having a positive impact on mental health. Some participants described suffering from depression in previous housing and that relocating to more suitable housing provided relief from their symptoms:

Housing does affect how you—you know, your health because the old house, it was dark, it was small. I was feeling really depressed—not the kids, me myself, I was feeling really depressed—but not this house. It’s bigger, it’s better; I’m happier.

—Tahira (female, Middle East, asylum seeker)

I had depression before, a good house had a positive impact on my mental health and I feel much better now.

—Parisa (female, Middle East, asylum seeker)

As such, while, participants’ levels of satisfaction with physical aspects of their housing was varied across interviews, all participants agreed that physical elements of housing (e.g., space, layout, heating and cooling and physical condition) impact health and wellbeing.

### 3.4. Social Environment

In addition to the physical features of a house, many participants noted that the social environment of their neighbourhood impacted the extent to which housing was experienced as suitable or otherwise and also influenced their broader resettlement experiences. In the interviews, participants emphasised several important aspects of their social environments, including safety and the presence of friendly and respectful neighbours, proximity to shops, public transport and school and proximity to their community, family and friends. The two key elements emerging from the findings, and discussed here, were safety and disorder, in addition to social connections.

#### 3.4.1. Safety and Disorder

The position in which a house is located is a key element of the impact that housing may have on health. In the current study, safety was a key concern for almost all participants, who suggested that safety and peace and quiet were essential elements of a neighbourhood, which if not realised, had the potential to negatively impact mental health. Specifically, many participants described feeling fear, worry, discomfort and uncertainty about how to deal experiences such as witnessing or being in proximity to crime (e.g., drugs, domestic violence) and excessive drinking, with some participants noting that they were unaware that they could call the police to seek assistance. For some new arrivals, staying in the neighbourhood became untenable. For instance, prior to relocating to her current neighbourhood, which has strong social bridges providing a feeling of safety and belonging, Hastee describes the atmosphere in her previous neighbourhood as one that created constant fear and anxiety. This was the family’s first experience living in the community after being provided emergency motel accommodation from a service provider:

Every two months we had police in our street and every morning we had police in front of the street. I saw in bus stop, people just transfer drugs and it was really bad because I had two boys and I was very worried for my son (…) I had stress all the time for my sons.

—Hastee (female, Middle East, asylum seeker)

Several other participants noted that they had experienced discrimination, threatening behaviour and been victims of crime. Edris, a Middle Eastern refugee, lives with his wife in supported accommodation and has experienced several break-ins.

The safety—you know—to feel safe. With the break-ins. This is not good. I want to feel safe. It (neighbourhood) impacts the mental—mental health. It is very important. It is an area that makes a big difference to you.

—Edris (male, Middle East, refugee)

Najme, an asylum seeker from Iran who has been living in the community for four months, had also been a victim of theft, which given her role as guardian to her niece and nephew, created a significant amount of worry:

I don’t like (the neighbourhood) you know, the old unit next to the housing and (you’re) not safe because the two times they’ve stolen the bicycles. We have to always worry about the door is closed or the car is safe; always worried about these things.

—Najme (female, Middle East, asylum seeker)

Participants also detailed experiences of threatening behaviour perpetuated by neighbours in the street. For example:

*I just worry about the guys, the people around [*the neighbourhood*] that want money (I scared) one day they kill me; that’s why I move from there.*

—Janan (male, Middle East, asylum seeker)

Both of these accounts speak to the heightened risks of experiencing threatening behaviour for asylum seekers and refugees. The impacts that these experiences have on the sense of wellbeing of the participants is further expressed by Rachel, a refugee from Southeast Asia, whose family experienced threatening behaviour from a previous neighbour who routinely approached their house in an intimidating fashion:

We frightened from the neighbour and then we also frightened from all Australians because of the neighbour. She was too—every day she was coming at our house and knocking and shouting (…) Like we lose plenty of confident after this.

—Rachel (female, Southeast Asia, refugee)

#### 3.4.2. Social Connections

The extent to which asylum seekers and refugees can experience successful resettlement and integration also relies on the availability of diverse social relationships, not only social connections (social bonds) within their own communities, but social ties to other communities (social “bridges”). In relation to housing, this may be particularly seen in having “good” neighbours. For example:

So the most important thing for me is the security and peace of mind. Yeah, security, safety and peace (…) good neighbours.

—Rasul (male, Middle East, refugee)

I mostly like it here because of the peace of mind I have here (…) because of the residents. It has good residents.

—Piruz (male, Middle East, asylum seeker)

However, there was evidence in some of the interviews of difficulties in establishing social bridges with people from other communities living in the same neighbourhood, often due to differences in cultural expectations and the absence of English skills that might otherwise facilitate stronger social connections. For example, in addition to his concerns around poor housing condition and affordability for new arrivals, Samuel speaks of the lack of neighbour relations in his area both in terms of lack of friendship, but also in terms of keeping an eye on each other for safety:

That’s a problem because almost I lost my confidence as a community to live together with the neighbour people to live together. I lost the confidence; that’s a very bad thing for me.

—Samuel (male, Africa, refugee)

For many participants, good neighbour relations were seen as important to health and wellbeing both in terms of establishing social bridges and in terms of garnering support and a sense of ontological security in their neighbourhood. While some participants were unable to establish those social bridges with their neighbours, others were the recipients of greater efforts towards social inclusion. Hastee, for example, details the social bridges established through the support of her landlord (also a neighbour) and the positive impact that this has had on her sense of safety and ontological security in her current neighbourhood, in contrast to her previous experience noted earlier:

(When) my landlord go for holiday and she just tell me …”you can contact (for example), number 13, they’re all home” (…) I went “oh that’s perfect. That’s for—anything happen I can go to every house. I know I’m safe here, opposite that house”.

—Hastee (female, Middle East, asylum seeker)

### 3.5. Insecurity of Tenure

Insecurity of tenure is a key characteristic of the private rental sector, particularly in Australia where standard leases are no more than 12 months [36]. This means that the vast majority of the participants in this study, who were in private rental accommodation, were subject to insecure tenure and the potential impacts of this for health and wellbeing. The health impacts described in this study were most often described as worry, fear of being displaced and the absence of peace of mind, and a number of the participants reported being concerned about their lease ending soon, landlords refusing to renew leases and difficulties finding another rental in a short time frame. Asylum-seeking males particularly noted a sense of insecurity in their shared housing arrangements:

Your house is a place for feeling relaxed and peaceful. If I rent I don’t feel good. I want to feel peaceful.

—Hozan (male, Middle East, asylum seeker)

I am worried about being displaced. It causes your mental and physical health to be endangered.

—Tirdad (male, Middle East, asylum seeker)

Maybe the owner of the house will say ‘I want to (turn) my house—or I’m giving notice, I need to repair this house.’ Where are we going to go with those kids?

—Ariane (female, Africa, refugee)

While all but a small number of the participants were experiencing insecurity of tenure in terms of being in rental properties, the circumstance of several African refugee families illustrated the profound mental health impacts of housing insecurity. At the time of the interviews, these families were being forced to move from their rental properties because the owners were planning to sell. Naeva, a single mother, for example, was experiencing significant stress in her attempts to secure housing for herself and the six children she had living with her. The family has occupied five houses during their nine years in Australia, and while they were provided with supported housing for the first five years, the following four years have seen them move between various private rentals. One of the woman’s daughters, who was translating her mother’s experiences in an interview, describesthe impact that the family’s precarious housing situation has had:

She (mother) doesn’t really like the fact that we move almost every year. She just wants to settle in, stay around the area so the kids not move all the time (…) And do you know, if you are moving (a lot) it can give you like stress, depression, moving. Even kids always complain ‘why are we always moving? Every time we miss our friends at school’ and whatever.

—Naeva (female, Africa, refugee)

Daina, another African refugee details a similar situation:

I worry a lot because like in this situation we haven’t found a house yet but we know that the—because we were told if we don’t move out when we are supposed to they will throw our things out so that means we could become homeless and that worries me a lot.

—Daina (female, Africa, refugee)

Daina has lived in her current housing for six and half years with her husband and their seven children. The family is experiencing a range of ongoing issues relating to the poor condition of the house and the unsuitable layout. During their time in this house, Daina and her husband have been unsuccessful in their attempts to find another more suitable house because of the lack of affordable housing with enough space for nine people. Recently, they have been told to look for another house because the owner plans to sell the house in which they are currently living. The difficulties that Daina has experienced with trying to find housing in the past are causing enormous worry and stress, and the risk of homelessness (the extreme of housing insecurity) is her greatest fear.

## 4. Discussion

In this section, we discuss the housing issues identified by the participants and the impacts of these issues on health and wellbeing. We reflect on how the findings relate to the models of Mallet and colleagues’ characterization of the health-housing interface, as well as how this sits within the broader integration framework of Ager and Strang. Finally, we discuss the strengths and limitations of the study and conclude with considerations of the implications of the findings for policy and practice.

As Phillimore and Goodson [27] (pp. 315–316) reflect: “(f)or those seeking refuge, it could be argued that the importance of finding a home is particularly symbolic as it marks the end of a journey and the point at which refugees can start to consider their wider need.” Overall, our research findings suggest that refugees and asylum seekers may form part of the “hidden fraction” [36,40] who experience significant problems in the Australian housing environment; for these individuals, the journey to safety and stability does not feel quite at its end. The findings also reflect the pathways through which Mallett and colleagues suggest that the housing people live in can impact health, specifically in relation to housing affordability, suitability and security and the intermediate factors involved. Our analysis found that participants experienced issues in relation to each of these areas and across multiple elements and, moreover, that issues in these features of housing were all seen as having a negative impact on health, in particular mental health. We also found that housing experiences interacted with other elements of integration as highlighted by Ager and Strang. In this section, we explore these findings before considering the implications of the research.

In particular, we found that participants discussed housing affordability as a key issue impacting their health (and particularly mental health), in addition to the cost of living in Australia more generally. Housing affordability has been linked to mental health [4,17,71,72], and general financial stress is a known mental health risk factor [73,74]. Issues with affordability were particularly acute for those with large families, as well as asylum seekers who in general have access to less financial resources. Housing affordability interacted with other indicators of integration such as language and employment, where language difficulties and problems securing employment left less money for good housing and led to particularly precarious housing conditions for some participants. A range of previous literature [35,45,48,75] has noted affordability issues in housing for refugee participants, and our findings highlight the negative mental and physical health impacts associated with this issue for both refugees and asylum seekers living in resettlement countries.

In addition to affordability, physical aspects of housing such as cold and damp housing, or housing that was an inappropriate size or layout for specific groups of people (such as single, male asylum seekers and larger families) were seen to be negative for health and wellbeing. Previous research has likewise found similar findings more generally including dampness as a risk factor for respiratory illness, health issues due to cold and extreme heat and the mental health issues associated with noise disturbance, inadequate privacy and housing in poor condition [18,20,21,40,76,77,78,79,80,81,82,83,84]. The current study indicates that physical elements of housing are likewise problematic for the health of refugees and asylum seekers, with asylum seekers again being especially vulnerable to living in housing that was unsuitable in terms of the condition of the house due to their precarious visa and financial situation, as well as feeling they had little control over these issues. This lack of control (both real and perceived) is particularly problematic for health, since a sense of control is a social determinant of health in its own right [85,86]. As Ager and Strang’s model highlights with rights at the base of integration experiences, those on temporary visas are also more likely to experience vulnerability in other aspects of their lives in countries of resettlement.

Participants also discussed social aspects of housing as important for health, and this was especially the case for housing that promoted feelings of safety. Having trust and confidence in your neighbourhood community also contributes to feelings of belonging and a sense of ontological security (or a feeling of confidence, continuity and trust in the world) [87]. Housing that is perceived as safe (or based in safe neighbourhoods) has been noted in previous research as particularly important for health [40,88,89,90], and there is a large body of research linking a fear of crime more generally and poorer mental health [91,92,93,94]. As Bonnefy [40] (p. 414) notes, safe housing: “enables the development of a sense of identity and attachment—as an individual or as part of a family, and provides a space to be oneself. Any intrusion of external factors or stressors strongly limits this feeling of safety, intimacy, and control, thereby reducing the mental and social function of the home.” These issues are particularly heightened for people with asylum-seeking and refugee backgrounds, for whom feelings of safety may be particularly important in light of backgrounds of torture and trauma and ongoing experiences of insecurity and uncertainty in resettlement countries [11,46,47,95].

The other key social element of housing that people highlighted was that of interactions with neighbours, which can have an important effect on the extent to which people newly arrived in a country feel welcomed [96]. In our study, participants highlighted that living in neighbourhoods where they were able to develop positive social connections was a key pathway to good health outcomes. However, participants in our study also noted that they had experienced discrimination in their neighbourhoods, most noticeably in relation to discriminatory behaviours from neighbours themselves, also leading to negative mental health outcomes reflecting a large body of research linking discrimination to ill health [97,98,99,100].

Previous research notes that barriers to housing security are common among people from a refugee background (UK [5,101]; Australia [2]), and insecure tenure is a well-known barrier to health, wellbeing and social inclusion [36,102,103]. Concerns about housing tenure were highlighted in this research where the majority of people were housed in the private rental market, which is characterised by short-term leases in Australia. The housing support system in Australia for refugees and asylum seekers also means that in addition to the likely involuntary move from their country of origin, people have generally previously had to leave housing involuntarily in Australia when their initial six months (six weeks in the case of asylum seekers) of provided accommodation finishes. In some cases, our participants had moved to suburbs that were geographically distant from their initial accommodation placement, with this move disrupting social networks and access to services, and this change may further heighten the negative effects of insecurity and a sense of a lack of control. Difficulties finding housing and concerns about housing tenure were particularly salient for our participants who were part of large families, who face additional barriers in securing housing [104].

In terms of the models of housing we discussed in the Introduction, our research indicates the value of Mallett and colleagues’ characterisation of precarious housing and the notion of cumulative housing issues, where most participants were experiencing issues in two or more elements of their housing, indicating that their housing was precarious. In addition, we found interactions between factors of integration noted by Ager and Strang, which in turn can compound (or ameliorate) people’s housing experiences. Our findings highlight in particular interactions between the domain of social connections and housing. This is the case both within and between communities (bonds and bridges respectively), which played an important role in influencing positive functional outcomes for asylum seekers and refugees, as well as having a positive impact on their health and wellbeing [105], linking housing and health. Further, in terms of Ager and Strang’s argument that rights and citizenship are the foundation for integration, our research indicates that being on a temporary visa affects all elements of the other domains, including the links between housing and health; for example, where those in poor housing that damages their health do not feel they can change this due to the constraints associated with their visa and their social status (perceived or otherwise) as asylum seekers in a resettlement country.

Overall, the study highlighted the importance of housing for the health and wellbeing of asylum seekers and refugees through a range of pathways outlined above. While the study included both refugees and asylum seekers from a range of cultural backgrounds and housing situations, the qualitative nature of the study means that the findings may not be generalizable across other groups of new arrivals or resettlement countries. Similarly, semi-structured interviews may also raise questions of generalizability, particularly in relation to the extent to which participants discuss issues of personal rather than more general importance, and the validity of the interview data in terms of the coverage of participants’ actual experience (e.g., participants may forget to mention, or deliberately omit, issues) [106]. However, the extent to which the themes were present across interviews and participants suggests that the housing issues and pathways to health impacts noted in this study are likely to impact refugees and asylum seekers across resettlement countries with similar housing stock and government support structures as Australia. Moreover, the range of participant demographics included in the study allowed us to note both specific housing needs (e.g., in relation to single males or large families), as well as more general issues experienced across our sample.

In relation to other limitations, the study asked people’s perceptions of the links between housing and health, rather than being able to directly measure either the nature of the housing itself, nor to directly assess the relationship between this and health using health measures. We focus here on the way that housing can affect health; however, there are clearly ways that health may influence both the nature of housing (e.g., poor health leading to more limited employment opportunities and money to secure better housing) and the perception of housing (where poor mental health may negatively influence perceptions of housing). Our sample included refugees and asylum seekers from a range of countries, which enabled a broader consideration of the links across country of origin, but means, given the limited sample size, that it was difficult to assess some of the particular cultural differences that may be relevant, which would require a larger sample. Our sample was drawn from a larger study of the links between housing and health for refugees and asylum seekers. It may be that those who initially agreed to participate in the study, and then the interviews, were those who were less happy with their housing. Alternatively, participants who did not participate may have been more marginalized, for example in terms of their English language skills or access to transport, which may also reflect more difficult housing circumstances.

Previously, we outlined the eight goals highlighted by the WHO [12] in relation to addressing housing as an SDOH, and these are all relevant in light of this research in terms of what is still required for people with refugee and asylum-seeking backgrounds in housing in resettlement countries. Some of the housing that people lived in was of poor quality and lacking in facilities such as heating and cooling (housing should have sound construction and offer basic services such as clean water and sanitation, and provide protection from climate changes), was crowded (be of an adequate size), left people feeling unsafe (provide safety and security), was too expensive (be reasonable and affordable), far from social connections and services (accessible to services and amenities) and insecure (offer reasonable security of tenure). Addressing each of these elements is likely to improve the health and wellbeing of people from refugee andasylum-seeking backgrounds.

In terms of practical recommendations, a key issue to emerge from our research is that of the impact of housing insecurity on the health and wellbeing of people with asylum-seeking and refugee backgrounds. In this regard, policy changes to improve housing security and reduce mobility for refugees and asylum seekers would improve health outcomes. For example, ensuring that both asylum seekers and refugees are eligible for social housing (this is not the case in South Australia) would lead to greater housing security, and this supports findings of research concerning housing and health for refugees in Canada, which found positive benefits to social housing availability [46,47]. Similarly, supporting pathways to home ownership would be valuable, as would increasing time limits on initial supported housing options. A greater diversity of housing being made available to refugees and asylum seekers both in terms of tenure type and other features of housing such as size and layout would better meet the various needs of different groups (e.g., large families, single people). While there are housing standards regulating housing in South Australia, and Australia more generally, these standards focus more on the physical aspects of housing, without consideration of the broader elements of housing that affect health, nor do they focus specifically on the needs of refugees and asylum seekers. A great consideration of these other aspects of housing and the needs of particular population groups would strengthen the effectiveness of these regulatory mechanisms to promote healthy housing.

Efforts to improve housing affordability, particularly for housing in good condition, such as granting full government benefits to asylum seekers, as well as raising social security benefits for refugees and asylum seekers more generally (recent reports indicate for those on these benefits that almost no properties are affordable [107,108]) would assist in improving health and wellbeing for asylum seekers and refugees. Participants in our study also noted that they felt that they could not address housing issues due to their refugee or asylum seeker status and also highlighted issues in relation to feelings of safety in neighbourhoods. Both of these issues could be addressed in part by greater understandings of the impact of torture or trauma for those working with this cohort of people (such as trauma informed training for real estate agents), ensuring that initial housing placements are in safe neighbourhoods, and making sure that people understand how to address behaviours that make them feel unsafe (e.g., processes for calling the police, access to interpreters to raise housing issues). In general, ensuring that service providers and policy makers are aware of the broad health impacts of housing on refugees and asylum seekers, particularly in relation to mental health, is key to improving health and wellbeing, as well as broader integration and resettlement outcomes for this group of people in countries such as Australia.

## 5. Conclusions

Overall, our research provides further evidence concerning the importance of housing to both physical and mental health for asylum seekers and refugees living in resettlement countries. Housing should be considered an important health issue when planning resettlement, and improving housing quality, affordability and increasing diversity in the type of housing available to asylum seeker and refugee families all have the potential to lead to more positive health outcomes and successful integration.
